# Perceptions and utilization of primary health care services in Iraq: findings from a national household survey

**DOI:** 10.1186/1472-698X-11-15

**Published:** 2011-12-16

**Authors:** Gilbert Burnham, Connie Hoe, Yuen Wai Hung, Agron Ferati, Allen Dyer, Thamer Al Hifi, Rabia Aboud, Tariq Hasoon

**Affiliations:** 1 Johns Hopkins Bloomberg School of Public Health, Center for Refugee and Disaster Response, Baltimore, MD USA; 2 International Medical Corps, Washington DC, USA; 3 Al Kindi College of Medicine, University of Baghdad, Iraq; 4 College of Medical and Biological Technologies, University of Baghdad, Iraq; 5 International Medical Corps, Baghdad, Iraq

## Abstract

**Background:**

After many years of sanctions and conflict, Iraq is rebuilding its health system, with a strong emphasis on the traditional hospital-based services. A network exists of public sector hospitals and clinics, as well as private clinics and a few private hospitals. Little data are available about the approximately 1400 Primary Health Care clinics (PHCCs) staffed with doctors. How do Iraqis utilize primary health care services? What are their preferences and perceptions of public primary health care clinics and private primary care services in general? How does household wealth affect choice of services?

**Methods:**

A 1256 household national survey was conducted in the catchment areas of randomly selected PHCCs in Iraq. A cluster of 10 households, beginning with a randomly selected start household, were interviewed in the service areas of seven public sector PHCC facilities in each of 17 of Iraq's 18 governorates. A questionnaire was developed using key informants. Teams of interviewers, including both males and females, were recruited and provided a week of training which included field practice. Teams then gathered data from households in the service areas of randomly selected clinics.

**Results:**

Iraqi participants are generally satisfied with the quality of primary care services available both in the public and private sector. Private clinics are generally the most popular source of primary care, however the PHCCs are utilized more by poorer households. In spite of free services available at PHCCs many households expressed difficulty in affording health care, especially in the purchase of medications. There is no evidence of informal payments to secure health services in the public sector.

**Conclusions:**

There is widespread satisfaction reported with primary health care services, and levels did not differ appreciably between public and private sectors. The public sector PHCCs are preferentially used by poorer populations where they are important providers. PHCC services are indeed free, with little evidence of informal payments to providers.

## Background

The 2003 US-led invasion occurred when the Iraq health system was already weakened from 23 years of dictatorship, the 1980-88 Iran-Iraq war, the 1990-91 Gulf War, and 12 years of embargos and sanctions [1]. The well-developed, hospital-centered health system, existing from before Saddam Hussein, had badly deteriorated by 1997, with a substantial decrease in services it could provide [2-4]. Following liberalizing of restrictions on drugs and supplies by the UN Security Council in 1996, there was some improvement in services and a slow start to reorientation toward primary health care services.

Soon after the invasion, substantial damage to the health sector occurred from widespread looting and destruction of facilities [5]. Early efforts to strengthen health services by US-led response were paralyzed by a mixture of inter-agency conflicts and political agendas [6]. By 2007, perceptions were that health services were continuing to deteriorate in physical condition and to be short of medicines [7]. As the conflict stretched on from 2003, medical doctors began migrating within Iraq as well as leaving Iraq for neighboring countries [8,9]. Both the ongoing violence and the construction of barriers limiting travel in violence-affected areas have restricted patient access, especially in Baghdad. During the worst violence, doctors minimized their exposure in health facilities, remaining in the relative safety of their homes for much of the time [10] Patients feared to venture out for treatment, and were often unable to secure critical drugs such as insulin [11]. With the migration of experienced doctors from Iraq, there have been concerns about the quality of health services, and the ability of training facilities to replace those migrating, especially those with advanced specialty training [12,13]. Those doctors remaining work in a health system that is heavily centralized, politicized and functioning in a non-transparent, non-accountable manner [14].

Most donor attention concerned with rebuilding health services in Iraq has focused on problems of hospitals. The interest in primary health care services has come about mainly in the past two years [15]. In Iraq there are about 2200 Primary Health Care Clinics (PHCCs), most located in population centers, but some in rural and peri-urban areas. Slightly more than 1400 PHCCs are staffed by doctors. Previously, some, though not many clinics were managed by nurses. Given the acute shortage of nurses in Iraq, subsequent to this study, almost all remaining nurses were reassigned to hospitals, leaving staffing of many facilities entirely in the hands of medical auxiliaries [16]. In some PHCCs doctors work alone, while other facilities may have up to 20 doctors. The number of doctors present is not necessarily based on workload or need, but more commonly on where doctors wish to live and the proximity of the PHCC to their house or their own private clinic. The difficulty with transportation, curfews, and numerous check points exacerbates this practice.

To understand how primary health care is perceived and utilized by Iraqis, we carried out a national cluster survey involving 1256 households. Questions focused on perceptions of quality, trust, costs of services, accessibility, utilization, and satisfaction with services received. Since our concern was generating information to assist the Iraqi Ministry of Health in responding to patient perceived needs at PHCCs, this survey was carried out in the catchment or service area of public sector clinics across Iraq. As health systems reconstruction efforts expand to include primary health services, it is important that efforts also consider the perceptions of health care seeking behaviors of the clients, rather than focusing primarily on structural issues.

## Methods

To specifically understand the perceptions and experiences of households with their local PHCC facilities, a cluster of 10 households were surveyed in the catchment areas of 126 clinics randomly selected from the national list of all doctor-staffed PHCs in Iraq. Seven facilities were selected per governorate regardless of number of facilities in each governorate. Three random back-up facilities were selected in case initially selected facilities proved to be inaccessible or in an insecure area.. Field work was done by the International Medical Corps (IMC) with assistance from Al Kindi College of Medicine and the Iraqi Red Crescent Society. Assistance with study design and analysis of data was provided by the Johns Hopkins Bloomberg School of Public Health. Ethical approval was given by the Al-Kindi College of Medicine, Baghdad, and the Institutional Review Board of Johns Hopkins Bloomberg School of Public Health declared the analysis of data as exempt.

The catchment or service area of a PHCC facility was defined as the households within two km of the PHCC. In almost all areas, streets were laid out in a grid pattern. GPS units are still not acceptable in Iraq, so a house selection process was used that first selected a random direction from the health facility. Using a three-digit randomly generated number ranging from 0 to 359, a direction from the PHCC was chosen. If the direction selected did not lead toward a residential area, the step was repeated. As the next stage, a two-digit number was randomly generated, with the first digit taken as the number of the crossroads to pass in the indicated direction, and the second digit as the number of households on the selected crossroad to pass in order to reach the start house. Once selected, the start house and the nine nearest consenting households were visited. At the household, the husband, wife, (or female head of household) mother-in-law or other resident adult was interviewed. If present, the husband would almost always be the household respondent. Expenditure data on health were collected only from the head of household. Where none of these were present or participation declined, the survey team continued to the nearest adjacent house until 10 households had been selected. Information was recorded on paper and sent to Baghdad for data entry and preliminary analysis. Subsequent analysis was completed in Baltimore using Stata statistical software version 11 (StataCorp LP, College Station, TX.) Statistical methods for complex survey data were applied to account for cluster sampling. To adjust for unequal selection probabilities, data were weighted by the number of health facilities per governorate.

Questions asked of households included household characteristics, details on any pregnancies, recent illnesses, where care was sought, satisfaction with services, costs and affordability of services, and household expenditure on health. Issues of health services trust were approached by asking households what services they would recommend to others for various childhood and adult conditions. Other questions concerned trust in the skills of the providers. As a proxy for wealth, the presence of specific household assets were queried, based on information from formative work. Among these were television, mobile phone, automobile, motorcycle, computer, generator, air conditioner and refrigerator. In the analysis, the presence of these were graded into three categories. Numbers of rooms and house construction were also recorded. The questions in this survey built on previous work with household surveys in Afghanistan, and among Iraqi households in Jordan and Syria [17,18]. The studies among Iraqis in Jordan and Syria looked at access, utilization and affordability of health services to refugee households, as well as illness patterns. For this current PHCC catchment area survey, the sample size was calculated on estimated childhood illness in the past two weeks. Allowing for a design effect of two and for refusal rates of 5% a sample size of 900 was judged adequate, using a power of 95% and precision of 80%.

## Results

### Demographic findings

In all, 1256 households, representing 7273 persons, were surveyed during May 2010. The survey was completed in 17 of 18 governorates, the 18^th ^(Erbil) being dropped for logistical problems. Refusal rate was less than 5% of households. Of respondents, 65.3% were male. The mean age of household informants was 44.6 years. The average respondent had 10.3 years of education. The average household size was 5.8 persons. Some 86% of households sampled stated that a PHCC facility was the closest health facility to their household. Although many households used other services than the PHCC for health care, the average travel time to the PHCC for all households surveyed was about 12 minutes. Of household members in the survey, 66.4% had been patients at the PHCC closest to their house, and an additional 7.3% were familiar with that PHCC, though household members themselves had not gone there for care.

Household data showed that one out of six households had moved in the past five years, with moving for security was the leading single reason. When these household data were further analyzed, households that have moved in the past five years had no difference in utilization of health services, levels of satisfaction or costs paid for care than those which have been longer term residents of the community.

### Births

In about 17% of households there had been a birth in the past year, but only 3.4% occurred in the PHCC, the majority being in public hospitals. This is not surprising as only a small number of PHCCs have delivery facilities. Median cost of delivery was US$68.46. Private clinics were preferred for antenatal visits, followed by PHCCs and public hospitals. In all, 63% of women giving birth in the past year had attended antenatal clinics more than three times. After birth, 55.2% of infants had received care at the PHCC. Of children under five in the households visited, about 75% had immunization cards available. Review of cards showed 308 of 374 (82.4%) of children under 24 months to be fully immunized for age. Among children under 12 months, 112 of 159 (70.4%) were fully immunized for age.

### Health services received during last illness (all sources)

Among children under five, 31.3% had been sick in the past two weeks. Of the 88.6% of children under age five who had received treatment outside the home, 40.5% were treated in a PHCC and 52.6% at a private doctor's clinic. The most common complaints were diarrhea (21.2%), sore throat (22.3%) cough or difficulty breathing (17.6%) and fever (14.5%). Among children over age five, 91.9% received treatment outside the home for the last illness, and somewhat less than half (44.8%) were treated at the PHCC. Among children over age five the most common complaints were flu-like symptoms, sore throat, and fever. Adults who were ill in the past 2 weeks sought treatment outside the home in about 86% of cases. Among adults, 20.8% utilized the PHCC, with the private doctor's clinic the preferred site by most (60.1%). The median cost of a patient visit (all types of facilities) was approximately US$20, but there were a number of large costs for visits reported which raised the mean cost to around $60.00 per visit. For around half of visits (all ages) the cost of medical treatment was thought reasonable by the head of household. More agreed that treatment of children was more reasonable (< 5 yrs: 52.2%; > 5 yrs: 52.8%) than was the costs of adult treatment (43.6%).

### Satisfaction

**Figure 1:**
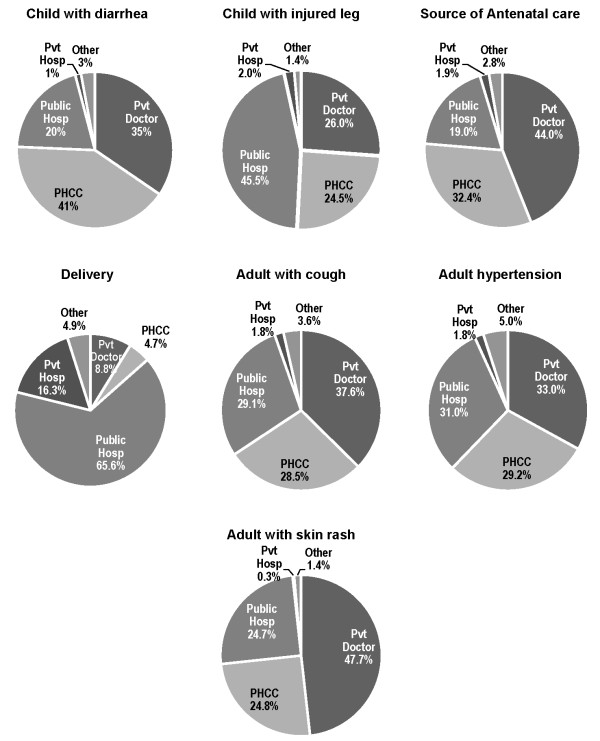
**Treatment locations recommended by householders for various conditions suggested**.

In general there was satisfaction with health care whatever the type of facility utilized (table 1), and this was for all age groups. About three-quarters of patients or caregivers happy with the services received during the last illness. Levels of satisfaction showed little difference between the reports for all PHC services combined (Table 1) and reports for PHCCs (Table 2). When indicators of satisfaction were compared between PHCCs and private facilities, there was little difference found. The PHCCs remained highly recommended for a number of conditions (Figure 1). The education level of the head of household had no affect on levels of satisfaction with services at various types of health facilities.

**Table 1:** Satisfaction with health care services (all sources) provided to adults or children (caretakers responding)

	Treated with courtesy	Waiting time too long	HW explained diagnosis clearly	Treatment not explained in a way we could understand	Full trust in skill of health worker treating us	Not told when to bring child back to clinic	Getting medicines was easy	Health unit not kept clean	Did not feel security was good at clinic	Over all satisfied with services we received
Child under 5 yrs*										

Strongly agree	144 (75.1)	46 (32.7)	87 (57.1)	21 (13.8)	81 (52.3)	50 (36.8)	75 (50.9)	31 (22.8)	23 (17.9)	71 (47.45)
Somewhat agree	26 (17.1)	68 (42.0)	29 (19.3)	29 (17.6)	38 (26.2)	22 (13.4)	27 (16.4)	49 (29.7)	25 (18.0)	42 (27.7)
Somewhat disagree	9 (6.7)	21 (13.8)	25 (16.7)	44 (30.7)	16 (10.6)	26 (19.5)	32 (21.6)	19 (14.8)	9 (6.9)	19 (13.2)
Disagree	2 (1.1)	15 (11.5)	9 (6.9)	55 (37.9)	15 (10.8)	44 (30.3)	17 (11.2)	50 (32.7)	74 (57.3)	17 (11.6)

Child 5-10 years*										

Strongly agree	96 (67.4)	47 (38.2)	63 (44.2)	17 (11.8)	71 (51.6)	62 (44.6)	56 (45.2)	20 (14.2)	16 (11.7)	57 (44.1)
Somewhat agree	36 (27.7)	63 (41.3)	40 (29.5)	34 (26.0)	36 (26.4)	26 (17.0)	36 (24.3)	46 (30.1)	17 (12.5)	37 (28.6)
Somewhat disagree	3 (2.1)	18 (12.8)	25 (19.8)	51 (36.7)	19 (14.9)	27 (22.1)	20 (14.8)	29 (24.2)	24 (19.9)	20 (14.8)
Disagree	3 (2.8)	10 (7.8)	8 (6.5)	34 (25.5)	10 (7.2)	21 (16.4)	22 (15.7)	42 (31.5)	75 (55.9)	16 (12.5)

***Adults***										

Strongly agree	226 (75.0)	104 (38.6)	159 (55.6)	57 (19.2)	168 (55.9)		132 (46.6)	73 (26.1)	65 (22.2)	147 (50.5)
Somewhat agree	58 (19.7)	107 (35.2)	67 (23.0)	66 (21.7)	71 (25.9)		69 (24.7)	63 (21.0)	44 (15.0)	83 (29.2)
Somewhat disagree	10 (4.3)	57 (18.8)	40 (15.4)	82 (28.6)	42 (14.0)		45 (15.5)	61 (21.5)	38 (12.9)	30 (10.3)
Disagree	4 (1.0)	25 (7.5)	18 (6.0)	93 (30.6)	12 (4.2)		39 (13.2)	90 (31.4)	146 (49.9)	30 (10.0)

*Only responses of caretakers who were present when services provided are included.

**Table 2:** Perception of respondents concerning the PHCC nearest to them

	I have trust in skill of doctors and nurses (%)	Waiting time is too long	The doctors and nurses treat people with courtesy	Medicines frequently not available	All treatments are free	Health workers are too rushed to understand your problem
Strongly agree	373 (53.7)	181 (26.4)	472 (67.6)	237 (34.1)	568 (84.2)	199 (30.2)
Somewhat agree	170 (24.1)	267 (38.2)	124 (19.0)	201 (29.1)	59 (7.9)	232 (33.8)
Somewhat disagree	79 (12.0)	139 (20.7)	63 (8.7)	154 (23.2)	40 (5.5)	136 (19.4)
Disagree	67 (10.1)	99 (14.7)	30 (4.8)	95 (13.7)	20 (2.4)	114 (16.7)

	The needed medical equipment is present and working when needed	The health unit is not kept clean	People in my neighborhood are satisfied with the care received at this clinic	People do not feel the security is good around the clinic	The hours the clinic is open are convenient	There are not enough female health workers to provide treatment for women

Strongly agree	156 (23.67)	166 (25.5)	294 (46.0)	124 (18.9)	394 (59.1)	258 (38.0)
Somewhat agree	201 (29.9)	130 (18.2)	183 (27.3)	76 (11.9)	110 (15.0)	131 (19.9)
Somewhat disagree	172 (24.9)	117 (18.1)	90 (13.6)	101 (15.0)	82 (12.6)	98 (15.9)
Disagree	148 (21.4)	271 (38.3)	89 (13.1)	369 (54.3)	91 (13.3)	181 (26.1)

This is restricted to users or those familiar with the PHCC.

### Local PHCC

Those respondents who had used their local PHCC or were familiar with its services by reputation, were asked about services provided there (Table 2). Trust in the skills of the doctors and nurses at the PHCC was high, with 77.8% saying they had some or full trust in the clinical staff. At the same time, about 64% felt clinic staff were too rushed to give their medical problems adequate attention. More than half reported waiting time was too long; this was a similar pattern of response for all health facilities, public and private. At the closest PHCC, 86.6% of persons responded that patients were treated with courtesy by the staff. For convenience of clinic hours, about 74% were somewhat or very satisfied. Medicines were felt to be generally available at the PHCC by only 37% of respondents. Almost all persons agreed that services provided in the PHCCs were free. There were 30.7% of patients who felt some degree of insecurity visiting the PHCC. There were 57.9% of respondents who observed there were not enough female health workers at that clinic, Of those making this observation, 68.5% were males. Nearly half observed that needed medical equipment at PHCCs was not always present or in working order. The PHCC was not thought to be kept clean by 43.7% of respondents.

### Cost of care

When households were analyzed by their asset levels, poorer households chose PHCCs for treatment more frequently than other sources both for children (p = 0.043) and adults (p = 0.044). Asset levels for larger families were no different from those for smaller families. The amount of money spent on health related costs by the household in the previous month was a median of 99,000 Dinars (US$77.02). Little of this was reported to have been spent at public sector facilities. Respondents stated that health services were free or that costs could be met out-of-pocket in 75.7% of households, but 16.9% had to borrow money in the last month to meet health costs, and 7.4% of households had to sell assets or still owed money for the most recent visit. There were no statistical differences in the amount spent for health in the past month between rich and poor households. As noted in Table 3, poor households more commonly reported that health care was too expensive (p = 0.001), and stated they had difficulty buying medicines (p = < 0.001). Persons with more education were more likely to say they could afford medicines (p < 0.001). Among women delivering in the past year, the number reporting more than three antenatal visits was related to higher household wealth status (p = 0.037).

**Table 3:** Perceived household access to care by wealth levels, measured by household assets

	Wealth level	Strongly agree	Somewhat agree	Disagree	Strongly disagree	Not sure	Totals	*P value*
We are able to get medical care whenever we need it	low	75 (35.4)	61 (26.1)	61 (27.1)	22 (9.7)	5 (1.8)	224	
	med	125 (34.6)	125 (34.5)	71 (20.2)	32 (9.4)	5 (1.3)	358	
	high	234 (38.4)	216 (31.4)	123 (17.9)	72 (10.4)	13 (2.0)	658	
	totals	434	402	255	126	23	1,240	0.2973

We cannot see medical specialists as often as is necessary	low	102 (45.9)	59 (24.1)	45 (21.7)	15 (7.3)	3 (1.0)	224	
	med	158 (44.9)	106 (29.3)	54 (16.3)	30 (7.9)	7 (1.6)	355	
	high	267 (41.2)	184 (28.1)	122 (20.0)	68 (9.7)	9 (1.0)	650	
	totals	527	349	221	113	19	1,229	0.6412

We can always afford to buy the medicine that we need	low	43 (19.0)	50 (21.2)	78 (36.0)	48 (21.8)	5 (2.0)	224	
	med	101 (29.3)	86 (22.5)	90 (24.7)	75 (22.2)	5 (1.1)	357	
	high	224 (36.0)	164 (24.9)	165 (24.5)	92 (13.6)	8 (1.0)	653	
	totals	368	300	333	215	18	1,234	0.0001

Medical care in the clinics is too expensive for us to afford	low	156 (72.0)	36 (15.9)	11 (4.8)	12 (5.5)	5 (1.8)	220	
	med	225 (65.5)	81 (23.7)	18 (4.6)	22 (5.9)	2 (0.3)	348	
	high	396 (62.3)	123 (20.7)	76 (11.9)	27 (3.8)	11 (1.3)	633	
	totals	777	240	105	61	18	1,201	0.0014

If someone from the family needs to be hospitalized, this will not produce financial difficulty for your household	low	145 (66.3)	42 (17.4)	17 (8.0)	13 (6.9)	4 (1.5)	221	
	med	220 (63.9)	78 (22.3)	32 (9.0)	9 (2.7)	7 (2.0)	346	
	high	393 (60.0)	138 (21.1)	70 (11.5)	33 (5.2)	15 (2.2)	649	
	Totals	758	258	119	55	26	1,216	0.4359

Asset classes for household wealth levels	low	Electric fan, kerosene heater, television, mobile phone						
	med	*Plus *air conditioner, generator, sewing machine, motorcycle, refrigerator						
	high	*Plus *automobile, computer						

In addition to household assets, the number of rooms present in a household were queried and the house construction noted by interviewers. On analysis, responses from people living in houses with fewer rooms, and with more basic house construction generally responded in the same way as seen with the asset scale. Analysis by asset grouping showed a greater correlation with health-seeking behavior than with the room or construction scales.

### Preferred treatment sites

Respondents were asked which facilities they would recommend for friends or relatives seeking care for a variety of common conditions (Figure 1). For all suggested conditions poorer households were more likely to choose the PHCC over other facilities, and this difference was significant for childhood diarrhea, child with an injured leg, adult hypertension, and an adult with a skin rash. However, for deliveries the more wealthy families still would recommend a public hospital (58.9%) over a private hospital (23.5%). The level of education of the head of household seemed to make no significant differences in the types of services recommended.

## Discussion

This household survey found there were few barriers to seeking health care outside the household in the Iraq. The large majority of Iraqi children and adults sick in the past two weeks had received care outside the household (86.0-91.9%). The high utilization is consistent with Articles 30 and 31 of the Iraqi constitution guaranteeing the right of all citizens to health care [19].

Respondents were generally satisfied with primary health care services available from public and private sources, and reported trust in health workers is high. The majority of respondents felt patients were treated with courtesy. Around a third of persons expressed some concern about the security situation when seeking health care services. When analyzed separately from all services combined, the pattern of satisfactions with public sector PHCC and other sources of primary care were generally similar. However, high satisfaction may in reality reflect low expectations from health services. In clinic exit interviews (IMC, unpublished) patients stated they did not expect doctors to communicate instructions for taking medicines, potential adverse effects, or information about their illness as "doctors have no time for this."

Almost all of those interviewed were aware of the services of the public sector PHCCs. These clinics were the site of treatment for the last illness in about 40% of children but in only 21% of adults. The remainder used private clinics and to a lesser extent public hospitals. Women delivering in the past year preferred private clinics for antenatal services, but most delivered in public hospitals. Only about 5% of the surveyed households had deliveries which occurred outside the formal health sector. This is far less than earlier national data have reported. **[**20] Many Iraqis feel that delivery in a private hospital frequently results in delivery by cesarean section. Few PHCCs have delivery services, which accounts for the three percent of pregnancies that were delivered here. Of children born in the past year, 55%, received their initial care in PHCC clinics.

Immunization coverage was found to be complete for age for measles in 69% of children 23 months and under and 65% for DPT3 by immunization card. While this is lower than reported values for Syria, Iran and Jordan, it does suggest that immunization has been relatively effective, despite on-going conflict [21].

Respondents were asked what type of services they would recommend to others for a variety of childhood and adult conditions. Although many would recommend the PHCCs for most of conditions suggested, for no conditions would more than half of respondents recommend PHCCs. Private doctors' offices would be recommended for 26-48% of outpatient conditions.

Of respondents who reported that they used or knew their local PHCC well, 63% reported that medicines were frequently not available. At their local PHCC, 64% felt health workers were too rushed, perhaps reflecting the general loss of Iraqi doctors from death and immigration. A shortage of female health workers at the local facilities was noted by 60%. In general, residents of PHCC service areas were satisfied with services, and felt they were treated courteously by competent health workers. The data suggest that PHCC facilities were well respected in the community. Traditionally, the health services in Iraq have been very hospital focused, with limited investment in primary health care. However, recent efforts have been made by the Ministry of Health and USAID to improve the quality of primary health care in Iraq [22].

Using household asset indicators it becomes evident that poorer households utilize PHCC services more than richer households. This emphasizes the role of PHCCs in providing the guaranteed right to health for all Iraqis, as set out in the Iraqi constitution. The PHCC services were in fact free, with few costs being reported for direct services. Poor households were no more likely to feel that hospitalization would produce financial difficulties than rich households. This also suggests that informal payments were not being extracted for hospital care. When free medications were not available from public facilities, as appears to be common in PHCCs, 57.8% of poorer households felt that they would have difficulty affording medicines, compared with 38.1% of richer households. The survey found expenditure on health in the past month was equivalent to $US77.20 which compares with $US50 found four years earlier in the large Iraq Family Health Service (IFHS) [20]. While our data would include some inflationary cost, it is likely that access and wealth of our catchment area population differed significantly for national values recorded four years earlier, perhaps being more urban than the IFHS sample. A slightly smaller percent of the 2010 study population could meet outpatient costs out of pocket than in 2006/7 (75.7% vs 86.1% in 2010), the significance of this is not clearly, though the 2010 study population being almost entirely urban or peri-urban, with the previous study having a greater rural population.

Overall, 15% of Iraqi population was thought to have been displaced within Iraq by 2008, using the International Organization for Migration estimates [23]. These estimates are very similar to the 14.2% of the population in this study (2010) who reported moving in the past five years. Not surprisingly, the majority of these moves were because of insecurity. Our 2010 migration findings could be an underestimate, as Erbil, a common destination for displaced Iraqi minorities, was not included in this survey for logistical reasons. Displaced populations are often marginalized from health care services, so it is reassuring to find no differences in utilization among the displaced living in the service area. Neither were there any differences in household assets from people who had moved into the area within five years.

Measuring the community perceptions of health services as reported here has an advantage over the commonly used health facilities exit interviews, which can suffer from a "gratitude bias" where patient satisfaction perceived health worker performance may be overstated [24,25]. While this household survey approach may allow a more settled reflection on the most recent consultation and incorporation of individual outcome indicators, there is a risk that recall bias may blur some details of the most recent visit. However, an advantage of the survey approach can be the capture of overall impressions, potentially based on multiple visits.

### Limitations

A survey of this nature has a number of limitations. Only households less than 2 km from the PHCC were included. Persons living further away from the health facilities may have different perceptions and utilization patterns than those living closer. Further, we included only those PHCCs staffed by medical doctors, excluding the large numbers managed by nurses and medical auxiliaries. Participants may also over-rate the value of services for fear of losing access to services, either individually or through health policy changes. In the analysis we included recent users with those having an acquaintance with conditions at the health facilities, though not recent users. This could dilute the results, but when analyzed separately there seemed to be little difference in the perceptions between these two groups. This survey has the potential biases of cluster surveys, collecting information from similar households and with insufficient cluster size to make comparisons among clinics and within governorates. However, population data were not available for a simple random sample. In the absence of objective PHCC performance data and interviews with professional staff, the user perceptions we recorded provides only part of the picture of primary care in Iraq. Nevertheless, user and community perceptions are key drivers of utilization practices, and are important for the planning of health services. Selecting from the catchment areas of PHCCs may have meant that low and middle income households are over-represented in the study, as public sector facilities are typically not sited in high income neighborhoods. Further studies to examine quality of Primary Health Care services from among a sample of various care sources could validate some of the observations reported here against objective criteria.

## Conclusion

The key themes from this survey are that PHCC facilities play a very important role in access to primary care health services in Iraq, and in general, user satisfaction is high, with little differences between public and private facilities. However, for many conditions, especially in adults and older children, the private medical clinics are more popular, especially as household income rises. The PHCC facilities are main sources of health services for poorer households and thus are a very important health asset in Iraq. There is a perception by users that health workers in the PHCCs are rushed, sometimes lack medicines, and working equipment. In spite of these concerns, communities adjacent to PHCCs have a high degree of satisfaction and trust for services provided. Their ostensibly free services indeed appear to be free, with few costs reported for direct clinic services. However, medications must be frequently purchased from outside pharmacies as they are not available at PHCCs. This appears to be an important barrier to treatment in poorer households. These clinics represent an important point of treatment in the otherwise hospital-focused Iraqi health system. These facilities can serve as a base for extension of primary health care coverage and community services in Iraq. Further investment in PHCC services could improve efficiency and effectiveness of the overall Iraqi health system.

## Competing interests

The authors declare that they have no competing interests.

## Authors' contributions

GB and TH developed the study design with assistance from AF, AD and TAH; TH, RA and YH oversaw data collection; CH led the data analysis; GB, TH and RA prepared the manuscript. All authors reviewed the final version of the manuscript. All authors read and approved the final manuscript.

## Pre-publication history

The pre-publication history for this paper can be accessed here:

http://www.biomedcentral.com/1472-698X/11/15/prepub
